# Update on the effects of microgravity on the musculoskeletal system

**DOI:** 10.1038/s41526-021-00158-4

**Published:** 2021-07-23

**Authors:** Otto J. Juhl, Evan G. Buettmann, Michael A. Friedman, Rachel C. DeNapoli, Gabriel A. Hoppock, Henry J. Donahue

**Affiliations:** Department of Biomedical Engineering, Virginia Commonwealth University, Richmond, VA USA

**Keywords:** Physiology, Medical research

## Abstract

With the reignited push for manned spaceflight and the development of companies focused on commercializing spaceflight, increased human ventures into space are inevitable. However, this venture would not be without risk. The lower gravitational force, known as microgravity, that would be experienced during spaceflight significantly disrupts many physiological systems. One of the most notably affected systems is the musculoskeletal system, where exposure to microgravity causes both bone and skeletal muscle loss, both of which have significant clinical implications. In this review, we focus on recent advancements in our understanding of how exposure to microgravity affects the musculoskeletal system. We will focus on the catabolic effects microgravity exposure has on both bone and skeletal muscle cells, as well as their respective progenitor stem cells. Additionally, we report on the mechanisms that underlie bone and muscle tissue loss resulting from exposure to microgravity and then discuss current countermeasures being evaluated. We reveal the gaps in the current knowledge and expound upon how current research is filling these gaps while also identifying new avenues of study as we continue to pursue manned spaceflight.

## Introduction

Roughly 60 years ago, Yuri Gagarin made history by becoming the first human to venture into space^1^. Since that time, international collaboration and technological advancements have allowed humans to venture deeper into space and stay for longer durations than imagined 60 years ago. Both human safety and health during space flight remain the greatest priority when considering further space exploration. Medical examinations done pre-, intra-, and post-spaceflight have demonstrated that exposure to microgravity, defined as forces less than 1 × 10^−3^ g, negatively impacts the human body and disrupts normal physiological systems, with none more affected than the musculoskeletal system^1–3^. Extensive research has been and continues to be done using in vitro and in vivo models to better understand the effects of microgravity exposure and develop countermeasures to the negative impacts incurred as a result of exposure to microgravity^3^.

The objective of this article is to review the current knowledge developed over the past decade regarding how real and simulated microgravity affects the cellular and physiological function of the musculoskeletal system. Based on current observations, it is clear that bone and skeletal muscle tissues are highly responsive and adaptive to their mechanical loading environment. However, more musculoskeletal system research is needed to understand how and why the pronounced bone and skeletal muscle mass loss in astronauts occurs in response to microgravity^1,2,4^. Moreover, we will focus on the multiple cell types of musculoskeletal tissues, along with their crosstalk interactions, and present potential countermeasures to alleviate the negative bone and muscle changes associated with microgravity. We will also discuss future avenues of research and gaps in our knowledge that need addressing as we venture deeper into space.

Bone is a complex tissue system that is constantly being remodeled throughout postnatal life to adapt to its mechanical loading environment^5–7^. This continually active process maintains the bone structure, mechanical strength, and function in response to the variable loading that we experience daily^2^. Under normal physiological loading, bone remodeling maintains homeostasis through bone-forming cells, osteoblasts, and bone-resorbing cells, osteoclasts. Exposure to microgravity disrupts this homeostatic balance and can result in up to a 1% loss in bone mass per month during continual microgravity exposure^8^. The balance between osteoblasts and osteoclasts is determined partly through osteocyte signaling. Osteocytes are mature osteoblasts that have become embedded within the bone matrix and comprise 90–95% of the total bone cell population^6^. Osteocytes are suggested to be the main mechanosensitive cell in bone and can detect mechanical signals acting on the bone tissue.

Although bone loss from aging is a common occurrence, the magnitude and speed of bone lost following exposure to microgravity is far greater than that which is typically seen during aging. This is a critical concern for humans when considering the effects of long-duration space flight^1–3^. Exacerbated bone loss leads to a decrease in bone mineral density (BMD), an increase in fracture risk, and a premature osteoporotic phenotype^3,9,10^. In this review, we will examine the physiological effects that exposure to microgravity has on osteoblasts, osteoclasts, osteocytes, and their progenitor cells and discuss countermeasures to block bone loss resulting from exposure to microgravity.

In addition to bone, exposure to microgravity also affects skeletal muscle. Skeletal muscles made up of individual muscle fibers generate force on our skeletons for movement and postural support. It is well established that skeletal muscle adaptation occurs in response to changes in its mechanical environment. For example, repetitive loading of skeletal muscle tissue leads to skeletal muscle hypertrophy whereas unloading of skeletal muscles under microgravity conditions leads to skeletal muscle atrophy^11,12^. This observed skeletal muscle atrophy due to exposure to microgravity is problematic because of its rapid onset and severity. Up to a 20% decrease in average skeletal muscle mass over 1 month and up to a 30% decrease in average skeletal muscle strength over 1 month can occur in astronauts exposed to microgravity^13^. This loss of skeletal muscle mass and function can hinder or prevent astronauts from performing mission tasks, such as performing spacewalks or necessary repairs on the international space station (ISS), as well as increase their risk of injury upon return to higher gravity conditions on Earth or Mars. Therefore, there is a need to better understand the cellular and molecular mechanisms of skeletal muscle atrophy caused by exposure to microgravity so new countermeasures can be developed.

Recently, the interaction between bone and skeletal muscle has become a field of interest in the musculoskeletal community^5^. Traditionally, bone and skeletal muscle tissue are studied independently, however, it has become apparent that to fully understand how the musculoskeletal system is affected by microgravity, bone and skeletal muscle must be studied together under standard (1 g) and microgravity (<1 g) conditions. It has also become clear that understanding the crosstalk between bone and skeletal muscle necessitates examining other physiological systems known to affect musculoskeletal health, such as the endocrine and nervous systems. These systems influence bone and skeletal muscle tissues under both standard gravity and microgravity. Understanding the interactions of these multiple systems pre-, intra-, and post- spaceflight is essential to gaining a complete understanding of how exposure to microgravity affects the musculoskeletal system. It also guides researchers as to what direction to take in future studies aimed at a better understanding of the effect of microgravity on bone and skeletal muscle.

Studying the effect of microgravity exposure on bone, skeletal muscle, or the interaction of both tissues has not been without challenge or expense. Therefore, as alternatives to using real microgravity, many ground-based analogs that simulate microgravity have been developed and characterized to allow further insight into the effect of microgravity on the musculoskeletal system. In addition, these approaches have led to the development of novel countermeasures to bone and skeletal muscle catabolism in response to microgravity.

## Models of Real and Simulated Microgravity

**Fig. 1:**
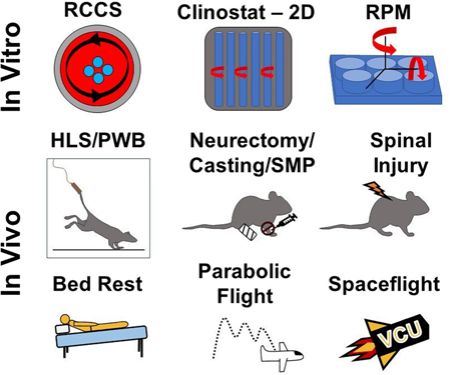
Models for in vitro, in vivo, and flight methods for inducing microgravity. RCCS, Clinostat, and RPM methods use rotation to create vector averaged forces to simulate microgravity. Hind limb suspension (HLS), spinal injury, and single limb immobilizationvia casting, neurectomy, or skeletal muscle paralysis (SMP) limit the ground reaction forces on the limbs by completely unloading or inducing disuse to simulate microgravity, while only HLS induces a cephalic fluid shift. Bed rest partially unloads the musculoskeletal system and induces a cephalic fluid shift when used in conjunction with head-down tilt. Parabolic flight uses rapid descent to reduce ground reaction forces and momentarily simulate microgravity. Real spaceflight removes ground reaction forces, mechanically unloading the sample or subject, and creates a cephalic fluid shift within the body.

Exposure to microgravity can be examined using both in vitro and in vivo models and exposure to either simulated or real microgravity conditions (Fig. 1). Typical in vitro models examine the influence of microgravity exposure on cells in and on constructs. Clinostats are tube-like structures that are filled with a cell suspension and rotate to simulate exposure to microgravity^1,14^. Important clinostat parameters include tube volume size and rotation speed which dictate the amount of centrifugal force needed to prevent driving cells toward the vessel walls^14^. Limitations of clinostats include tube width and smaller sample size and volume. Random positioning machines (RPM) are another type of device used to model and expose cells to simulated microgravity. RPMs consist of two motor-driven frames that allow cells and constructs to be freely rotated, simulating a microgravity environment via gravity vector averaging^14,15^. Limitations of RPM devices include the size and volume of samples in addition to the significant difficulty of introducing pharmacological agents or other experimental variables during rotation. The last commonly used in vitro model is the rotating wall vessel (RWV). RWVs are slow-rotating, liquid-filled containers developed and approved as a simulated microgravity model by NASA^14,16^. RWVs are also referred to as rotary cell culture systems (RCCS)^4,17^. Similar to the clinostat, RWVs rotate constantly. This rotation maintains a constant cell suspension, creating what is known as vector averaged microgravity, where all forces have an equal and opposite force which creates an overall net force of zero on the sample^14^. The speed at which this rotation occurs is determined is based on the specimen’s specific gravity, vessel fluid density, and viscosity. Since the devlopment of RWVs, alternate models have emerged with various vessel sizes and shapes, including the slow turning lateral vessel (STLV) and the high aspect ratio vessel (HARV)^4,17,18^. Although the models and devices mentioned above create an environment similar to microgravity conditions, it is important to emphasize that these devices only create a net zero gravity vector, rather than the reduced or near zero gravity experienced in real microgravity.

There are two commonly used in vivo microgravity animal models: hindlimb suspension (HLS) and skeletal muscle paralysis (SMP). HLS is a NASA-developed model that exposes the hindlimbs of a rodent to simulated microgravity through tail suspension^1,19^. This model causes musculoskeletal unloading of the hind limbs, and a cephalic fluid shift similar to that experienced by astronauts during spaceflight^1,19^. However, a limitation to HLS is that rodents often experience weight loss and increased markers of stress compared to ground control animals, making isolation of gravity-specific affects challenging. Partial weight bearing (PWB) is another model of suspension where the entire body of the animal is suspended, reducing the weight each limb bears. This method is favorable in that it can simulate more precisely the reduced gravitational loads experienced found on the Moon (0.16 g) or Mars (0.38 g). This method significantly reduces bone density and muscle mass^20,21^. Another model to induce musculoskeletal unloading is via SMP through the use of either Botox or a neurectomy to cause rapid musculoskeletal tissue loss in one of the hindlimbs. A benefit of the unilateral spinal cord injury model (neurectomy), is that atrophy occurs in both the skeletal muscle and bone tissue, as well as a disruption of the cardiovascular and immune systems, both of which are altered in astronauts during long-duration space flight^22^. Single limb immobilization via casting is a newer model that also causes skeletal muscle and bone loss similar to that experienced during exposure to microgravity environments^23^. However, this model is still being developed and few studies have been performed to evaluate the efficacy of this model overall. Limb immobilization models of paralysis via casting, Botox, neurectomy, and spinal injury along with PWB, however, do not create the cephalic fluid shift or entirely prevent bone loading from ground reaction forces observed in the HLS model. Although these models of simulated microgravity are an established alternative to exposure to real microgravity exposure, simulated microgravity only mitigates the ground reaction forces, and not the gravitational forces, experienced by the animals^24^.

The most clinically relevant method to examine the effects of microgravity exposure on the musculoskeletal system is through spaceflight. Unfortunately, experiments conducted in real microgravity are limited due to the infrequency of spaceflights, available capsule space, and extraordinary cost^15^. While examination of astronauts to evaluate the effects of microgravity on the musculoskeletal system has been performed, the information collected is limited to minimally invasive procedures and does not allow a full examination of the effects of microgravity on the musculoskeletal system. An alternate approach to examine the effects of microgravity exposure on humans is through the use of a head-down tilt bed rest model^1,25^. The head-down tilt bed rest model simulates microgravity by inducing musculoskeletal disuse and mimics the cephalic fluid shifts observed during spaceflight through the tilted incline. Microgravity can also be simulated during parabolic flights. Parabolic flights provide short-term freefall that simulate exposure to microgravity by transiently minimizing the ground reaction forces^26^.

## Hematopoietic and Bone Mesenchymal Stem Cells

**Fig. 2:**
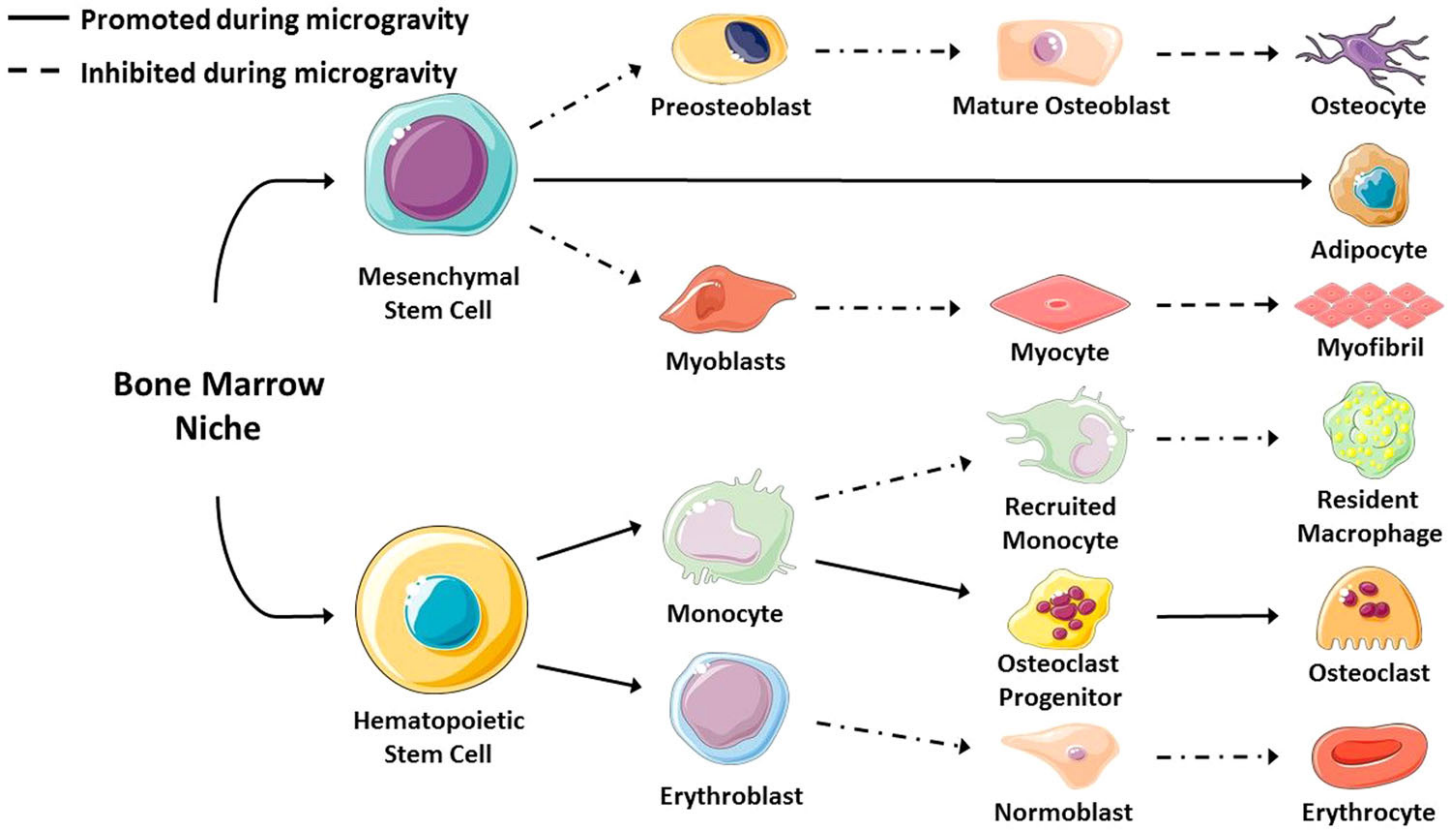
Schematic illustrating the effect of microgravity on the differentiation of stem cells into various cell lineages. Microgravity increases commitment to the adipogenic and osteoclastic lineage while decreasing commitment to the osteoblastic, myocytic, erythrocytic, and macrophage lineages. Created using images modified from Servier Medical Art by Servier.

Bone marrow, located in the interior of bones, is home to many different cell types, including bone marrow stromal cells (BMSC) and hematopoietic stem cells (HSCs), with vast differentiation potential as seen in Fig. 2^27–29^. Cells within the BMSC population give rise to mesenchymal stem cells (MSCs) which differentiate to bone-forming osteoblasts, cartilage forming chondrocytes, adipose forming adipocytes, or skeletal muscle forming myoblasts^30^. HSCs that differentiate into immune cells, erythrocytes, and osteoclasts are crucial for the repair of damaged bone tissue and bone turnover. BMSCs which differentiate into osteoblasts, osteocytes, myocytes, or adipocytes are critical for tissue regeneration and homeostasis.

Exposure to microgravity leads BMSCs to preferentially differentiate to adipogenic lineage cells as opposed to the osteogenic or myogenic lineage cells^31^. This decreases their capacity to maintain healthy bone and skeletal muscle tissue as well as to respond and adapt to the mechanical environment. Increased commitment of BMSCs to the adipogenic lineage also reduces the ability of bone and skeletal muscle tissue to regenerate and recover from injury. Additionally, unloading can decrease the population and function of BMSCs^32^. These effects are similar to what is seen with aging, and thus, studying the effects of exposure to microgravity can be useful for understanding the effects of aging.

### In vitro studies of the effects of microgravity on stem cell physiology

Data on how HSCs respond to microgravity is limited, as few studies have been conducted on HSCs in vitro. Mechanical loading increases HSC differentiation and maturation, suggesting that unloading from exposure to microgravity may prevent these effects^33^. Indeed, hematopoietic clusters of differentiation-34+ (CD34^+^) cells, when exposed to microgravity for 11 days or simulated microgravity for 2 days, have decreased proliferation and differentiation^34^. Microgravity also disrupts the migration potential and cell cycle progression of these cells^35^. Myeloid progenitor cell counts increase while erythroid progenitor cell counts decrease in microgravity. Differentiation towards macrophage lineage cells increases. Furthermore, simulated microgravity decreases erythrocyte proliferation and increases apoptosis which could have negative effects on tissue oxygen levels^36^.

Exposure to simulated microgravity decreases BMSC differentiation to osteoblasts which can have negative effects on bone formation and bone volume^37^. Simulated microgravity decreases BMSC cell proliferation, changes cell nucleus size, and reduces expression of cytoskeletal and musculoskeletal proteins^38^. Progression of the cell cycle is also disrupted, and the effects of growth factors such as insulin-like growth factor 1 (IGF-1) are attenuated^39–43^. These changes will likely lead to the decreased osteoblastic differentiation seen in microgravity. For example, collagen type I-alpha1 (*Col1a1*), a gene that is indicative of osteoblastic differentiation and increases as differentiation occurs, decreased after 7 days in simulated microgravity^44,45^. Additionally, phosphorylation of focal adhesion kinase (FAK) and proline-rich tyrosine kinase 2 (PYK2), which are required for osteoblastic differentiation and function, are decreased^46,47^.

Return to standard gravity after spaceflight restores bone volume but does not fully restore osteoblast number, indicating that microgravity has long-term effects on the capacity of BMSCs to differentiate into osteoblasts^28^. Culturing cells under pro-osteogenic conditions after exposure to microgravity is unable to promote osteoblastic differentiation as an expression of alkaline phosphatase *(Alpl)*, *Col1a1*, runt-related transcription factor 2 (*Runx2)*, and osteonectin (*Sparc)*, genes associated with bone formation, are decreased^37,45^. Meanwhile, expression of peroxisome proliferator-activated receptor gamma *(Ppar-γ*) (adipsin (*Cfd*), leptin (*Lep*), and glucose transporter-4 (*Glut-4*), genes associated with adipogenesis, are increased^31^.

### In vivo studies of the effect of microgravity on stem cell physiology

During exposure to microgravity, there is a decrease in the differentiation of HSCs into red blood cells^34,48^. Studies of astronauts in spaceflight and animals exposed to simulated microgravity revealed that exposure to microgravity induces a condition called space anemia^48,49^. This condition is characterized by a reduction in red blood cell mass, hemoglobin mass, and plasma volume. Trabecular bone loss during spaceflight also affects HSCs since hematopoiesis often occurs at locations of greatest trabecular bone volume^29^. Additionally, the accumulation of adipocytes and increased adipogenesis in the bone marrow after exposure to microgravity likely negatively affect HSC function based on the previous literature^50^.

In vivo models of simulated microgravity reveal similar effects of microgravity on BMSCs as in vitro models. Overall, human and rodent BMSCs are less osteogenic, differentiating less readily into osteoblasts during exposure to microgravity^45^. Mice that are mechanically stimulated by whole-body vibrations during exposure to simulated microgravity have an increased population of BMSCs, indicating exposure to mechanical loading during exposure to microgravity has a protective effect on these cells^28^. However, bone marrow cells of mice returning from exposure to microgravity during 2 weeks of spaceflight surprisingly show an increased differentiation potential upon return to normal gravitational conditions^51^. It is hypothesized that this occurs because exposure to microgravity hinders BMSC and HSC growth and differentiation, leading to an accumulation of undifferentiated cells and increased numbers of differentiated cells on return to normal gravitational conditions (1 g).

## Osteoblasts

Osteoblasts are derived from MSCs of the periosteum and bone marrow and are the primary cells that form bone. Throughout an osteoblast’s differentiation and maturation process, each osteoblast deposits the extracellular matrix and facilitates matrix mineralization. When an osteoblast becomes entrapped in the mineralized extracellular matrix it has produced, it will undergo terminal differentiation into an osteocyte.

Osteoblasts can both sense and adapt to changes in gravitational forces. Under microgravity conditions, changes in the loading environment cause variations in osteoblast proliferation, maturation, and activity and therefore changes in de novo bone formation^8,39,52^. Exposure to microgravity also results in changes in osteoblastic gene expression, cell morphology, and mineralization ability both in vitro and in vivo. However, specific mechanisms by which microgravity influences osteoblastogenesis remain poorly understood.

### In vitro studies of the effect of microgravity on osteoblast cell physiology

Under standard gravity conditions, temporal upregulation of pre-osteoblastic genes *Runx2*, *Col1a1*, and activator protein-1 (*Ap-1*), along with a host of other genes, indicates an increase in osteoblast differentiation. Mature osteoblasts then express, at high levels, *All*, osterix (*Sp7*), and osteocalcin (OCN; encoded by *Bglap*)^53^. These genes and associated downstream proteins are essential for bone formation, initiating the osteoblastic maturation and bone mineralization cascade. In cells exposed to simulated microgravity by RPM, Prado et al. identified 140 genes in 2T3 preosteoblastic cells that were differentially regulated compared to cells cultured in a standard gravity environment^54^. Genes associated with osteoblastic differentiation such as *Alpl*, *Runx2*, *Bglap*, *Col1a1*, bone morphogenic protein-2 (*Bmp-2*), and genes encoding various integrins, were all significantly downregulated after exposure to simulated microgravity^54^. This suggests that, due to microgravity, genes associated with osteoblastic differentiation and bone formation are significantly affected and downregulated. In support of these findings, aboard the Foton 10 spaceflight, MG-63 cells, an osteosarcoma cell line, also exhibited downregulation of genes encoding the proteins ALPL, OCN, and COL1A1 when compared to ground-based controls^55^.

In addition to the downregulation of key osteoblastic genes, Hu et al. observed that cells exposed to simulated microgravity displayed decreased mineralization^56^. This suggests that exposure to microgravity results in an overall decrease in BMD which is caused, in part, by a decrease in osteoblast number and differentiation during microgravity, which contributes to an imbalance of bone formation and bone resorption^54,56,57^. How variations in gravitational forces regulate and induce changes in the osteoblastic phenotype remains unclear, but evidence suggests a few possible mechanisms.

One possible mechanism underlying the effects of microgravity on osteoblast differentiation is the downregulation of ras homolog family member A (RHOA) protein. Changes in the cytoskeleton have hinted at a possible mechanism by which variations in the mechanical loading environment are transduced. RHOA regulates cytoskeletal arrangement through upregulation of Rho-associated protein kinase (ROCK) and the formation of actin fibers^58^. Along with regulating cell size and shape, evidence suggests that the actin stress fibers within the cell have a role in mechanotransduction, relaying mechanical signals to the nuclei and modulating cellular gene expression^58–60^. This evidence has led to more studies investigating the effects of microgravity on cytoskeletal structure, morphology, and composition. Most recently, Nabavi et al. exposed primary osteoblasts isolated from CD1 mice to microgravity for 5 days and observed a decrease in overall microtubule organization, a decrease in focal adhesion size and number, and a decrease in F-actin thickness compared to ground control cells^60^. Additionally, the nuclei of osteoblasts exposed to microgravity were larger and more irregular in shape compared to ground control cells^60^. This could indicate the onset of anoikis, a programmed cell death initiated by changes in nuclear morphology. This mechanically stimulated cell apoptosis provides a mechanistic explanation of the decreased bone formation seen during exposure to microgravity. However, in multiple studies, osteoblast apoptosis remained unchanged during exposure to simulated microgravity, though the osteoblasts’ sensitivity to apoptotic factors was drastically increased^61,62^.

Variations in intracellular Ca^2+^ concentration ([Ca^2+^]) have also been observed in cells exposed to microgravity. [Ca^2+^] is an integral second messenger within osteoblasts that regulates proliferation and differentiation^63,64^. [Ca^2+^] also plays an integral role in the regulation of cytoskeletal changes^65,66^. This relationship suggests that variations in [Ca^2+^] may, in part, be regulating changes in osteoblast phenotype after microgravity exposure. A study by Sun et al. observed decreases in [Ca^2+^] in primary mouse osteoblasts after exposure to simulated microgravity by clinorotation^65^. They also observed a decrease in voltage-sensitive Ca^2+^ channel activity and a functional decrease in the expression of both ryanodine and inositol 1,4,5-triphosphate receptors, which mediate intracellular Ca^2+^ release^65,67^. Additionally, studies by Michaletti et al. have shown significant changes in osteoblastic mitochondrion homeostasis and proteomic pathways after exposure to simulated microgravity using RPM^68^. Specifically, glycolysis, the Kreb’s cycle, the pentose phosphate pathway, the glycerol-phosphate shuttle, as well the malate-aspartate shuttle, were all significantly reduced by microgravity. Additionally, typical mitochondrial activity appeared dysregulated after simulated microgravity by RPM, which the authors suggest is an adaptive response to ensure sufficient cell energy during microgravity^68^.

Gap junctions also play a role in the response of bone to its mechanical environment. Gap junctions are membrane-spanning channels, permeable to signaling molecules less than 1 kD in size, that allow cells to communicate with one another^69^. Connexin 43 (Cx43) is the predominant gap junction protein in bone. Compelling in vitro evidence from our lab, and several others suggests that gap junction intracellular communication (GJIC) contributes to osteoblast and osteocyte mechanotransduction in bone. For instance, mechanical signals regulate Cx43 levels and GJIC between bone cells^70–76^. GJIC sensitizes bone cell networks to diverse extracellular signals^77–79^, and mechanically-induced signals are communicated between bone cells via gap junctions^80–82^. More recently Gupta et al. demonstrated that Cx43 affects both Wnt-dependent and Wnt-independent activation of β-catenin in osteoblasts^83^, both of which play important roles in the response of bone to its mechanical environment. Furthermore, Xu et al. found that in vitro simulated microgravity by RPM affects osteocytic Cx43 levels and gap junction hemichannel activity in a rather complicated manner^84^. Taken together these findings suggest that osteoblast and osteocyte Cx43 plays a role in the response of bone to microgravity. This is strongly supported by in vivo data described in the next section.

### In vivo studies of the effect of microgravity on osteoblast cell physiology

Osteoblasts show similar phenotypic changes in simulated microgravity in vivo as those observed during in vitro studies. In medaka fish exposed to microgravity aboard the ISS, a rapid and significant increase in osteoblastic genes *sp7* and *bglap* were observed 1 day after launch and continued for up to 8 days^85^. However, osteoblastic genes *sp7* and *col1a1* were downregulated after 60 days of microgravity exposure, but not significantly^86^. This resulted in a decrease in osteoblastic activity and a decrease in collagen within the bone matrix, but this was not consistently observed. Overall, these fish did display a decrease in BMD, but this was linked to a significant increase in osteoclast activity, suggesting that while rapid increases in bone formation genes may occur upon microgravity exposure, they quickly return to or decrease below ground-control levels^86^. Conversely, a study conducted in 23-week-old male C57/BL6 mice flown for 30 days during the Bion-M1 mission observed no significant change in osteoblastic activity, despite observing significant (64%) bone volume loss in the trabecular bone of the femur and decreased mechanical properties^87^. This finding is not typical, as other reports, which used younger mice and rats, showed decreased osteoblastic activity^37,87^. This highlights the importance of using skeletally mature animals in future studies of microgravity-induced bone loss to limit confounding variables such as bone growth and maturation.

Osteoblastic apoptosis may also contribute to microgravity-induced bone loss. During 7 days of unloading via HLS, an increase in osteoblast apoptosis and a reduction in the total number of osteoblasts was observed by histological quantification as rapidly as 2 days after unloading began^88^. Further, a marginal decrease in integrin α5β1 and a nonsignificant decrease in FAK phosphorylation was observed 2 and 4 days after HLS, respectively^88^. In addition to increases in osteoblastic apoptosis during microgravity exposure, decreases in osteoblastic activity was also evidenced by a twofold decrease in mineralizing surface of the tibial bone after 28 days of HLS^89^.

Another mechanism for microgravity-induced bone loss that has been proposed is an increase in cell quiescence/senescence. For example, during a 15-day exposure to microgravity on the STS-131 shuttle, female C57BL/6 J mice exhibited a roughly 6% decrease in bone volume fraction and 11% decrease in bone thickness^90^. Osteoblasts expressed a roughly threefold increase in cyclin-dependent kinase inhibitor 1A (*Cdkn1a/p21*) gene expression, a mediator of cell cycle arrest, and a roughly 1.5-fold decrease in cell tumor antigen 53 (*Trp53/p53*) gene expression, an inducer of cell apoptosis^90^. Numerous other genes that regulate the cell cycle and cell apoptosis were also differentially regulated. Increases in F-box only protein-4 (*Fbxo4*) and F-box only protein-31 (*Fbxo31*), which stimulate cyclin-dependent protein-1 (*Ccnd1*) and thus the transition from G1 to S phase, were observed as well as decreases in death domain-containing protein (*Cradd*), death-associated protein kinase-1 (*Dapk1*), HECT, C2, and WW domain-containing E3 ubiquitin-protein ligase 2 (*Hecw2*), and mitogen-activated protein kinase-10 (*Mapk10*), which are all apoptosis-related genes^90^. These data suggest that microgravity disrupts the osteoblastic cell cycle and that this may be partially responsible for bone loss, which is typically observed during microgravity exposure.

Gap junctions and Cx43 also play an important role in bone adaptation to unloading as would occur in microgravity. Our lab demonstrated that in mice Cx43-deficient bone is less sensitive to HLS-induced unloading^91,92^, and this involves both arms of bone remodeling^93^, while Grimston et al. showed similar results for muscle paralysis-induced bone loss^94^. Taken together these findings suggest that Cx43 deficiency may be protective against microgravity-induced bone loss and that Cx43 may be a novel countermeasure target for the prevention of bone loss due to microgravity. However, as mounting evidence suggests that osteocytes are the primary mechanosensors in bone the focus has shifted to evaluating the role of osteocytic Cx43 in microgravity-induced bone loss, as discussed later in this review.

Osteotropic hormones also contribute to the effects of microgravity on bone. For instance, in humans, bone-specific alkaline phosphatase (BSAP), a marker of osteoblastic differentiation, significantly decreased in early spaceflight (<14 days into a mission) but recovered to preflight levels during the remainder of the mission (60–110 days)^95^. Parathyroid hormone (PTH), a positive regulator of the Wnt-signaling pathway, calcitonin, and OCN, both osteogenic promoters, were all reduced for the duration of the flight, but again recovered upon return to standard gravity after spaceflight^95^. Evidence from the NASA Twin Study has shown that protein levels of both OCN and BSAP measured in urine are markedly increased in the twin that experienced space flight (<120 days) compared to the ground-matched twin. These levels decrease with long-duration spaceflight until the subject returns to standard gravity, and in time levels return to basal conditions^96^. Unfortunately, bone mass measurements were not reported in this study, making it difficult to determine the overall outcome of the data presented. Despite this, these data do suggest that after exposure to microgravity, the body adapts to the reduction in mechanical load by increasing levels of osteotropic factors associated with bone formation in an attempt to slow bone catabolism, a change not always readily observed in animal models.

## Osteocytes

Osteocytes are the most abundant bone cells, making up more than 90% of all bone cells^6,97^. Over the past few decades, evidence has emerged that osteocytes are the main mechanosensory cells in bone, although this concept is still developing^6,98–100^. Osteocytes are post-mitotic, terminally differentiated osteoblasts that have become encapsulated in mineralized bone matrix. Osteocytes express the genes encoding dentin matrix acidic phosphoprotein-1

(*Dmp1*), matrix extracellular phosphoglycoprotein (*Mepe*), and phosphate regulating endopeptidase homolog X-Linked (*Phex*) during early differentiation, which regulate proteins that aid in bone matrix formation and regulation. More mature osteocytes express genes encoding sclerostin (*Sost*) and fibroblast growth factor-23 (*Fgf23*), which regulate osteoblast activity and phosphate metabolism, respectively. Osteocytes have also been shown to be the predominant bone cell expressing genes encoding proteins for receptor activator of nuclear factor-kappa-Β ligand (RANKL) and osteoprotegerin (OPG) proteins, which are positive and negative regulators, respectively, of osteoclast formation^97,101^.

Each osteocyte has numerous dendritic processes that are connected to other osteocytes as well as osteoblasts and osteoclasts, thereby creating a vast communication network called the lacunar-canalicular system^6,100^. It is through this system that osteocytes are hypothesized to regulate bone homeostasis in response to bone loading or unloading, such as that seen during exposure to microgravity. As the main mechanosensory cells in bone, osteocytes can alter the bone formation and resorption depending on which proteins they secrete. Because of this, osteocytes have become a key focus in research evaluating methods to mitigate the bone loss caused by exposure to microgravity.

### In vitro studies of the effect of microgravity on osteocyte cell physiology

In vitro examination of the effects of exposure to microgravity on osteocytes was enabled by the development of cell lines, including MLO-Y4 and OCY454, that possess characteristics of primary osteocytes^102,103^. These characteristics include dendritic morphology and expression of osteocyte-specific genes^102,103^. Two caveats that must be considered are that, under most experimental conditions, these cell lines are not encased in a three-dimensional mineral matrix. Additionally, these cells undergo proliferation in vitro whereas available evidence suggests that in vivo osteocytes do not proliferate. Despite these caveats, examination of OCY454 and MLO-Y4 cell lines has greatly enhanced our understanding of osteocyte biology

Studies using OCY454 have demonstrated that the expression of *Sost* and *Tnsrf11*, which encode RANKL, are upregulated and expression of the gene encoding OPG is downregulated after exposure to simulated microgravity using an RCCS^103,104^. These findings demonstrate that osteocytes directly respond to microgravity by expressing genes that promote factors that promote osteoclastic differentiation while simultaneously inhibiting osteoblastic differentiation. In addition to examining specific genes that are differentially regulated during exposure to microgravity, in vitro studies are better suited to determine the mechanisms by which these changes occur. For example, deletion of *IGF-1* resulted in a decrease in the osteocytic response to mechanical stimuli^105^. Other in vitro studies have investigated the role of Cx43 in MLO-Y4 cells exposed to simulated microgravity. Previous work has shown Cx43 is necessary for Prostaglandin E_2_ (Pge2) release, a key hormone affecting osteoblast and osteoclast activity^106^. During exposure to microgravity by RPM, Cx43 deficient MLO-Y4 osteocytes show attenuated Pge2 release, altering bone metabolism^84^. This same study showed increased Cx43 hemichannel activity in response to simulated microgravity, implying increased intracellular communication.

### In vivo studies of the effect of microgravity on osteocyte cell physiology

Most, if not all, in vivo studies that exposed animals to microgravity, observed a decrease in bone mass. For example, a study examining the iliac crests of monkeys that were exposed to microgravity for 2 weeks during the Bion-11 space mission found an increase in collagen protein biosynthesis in osteocytes^107^. This increase resulted in the bone not undergoing the same extent of mineralization that is normally seen under standard gravity. This decrease in mineralization, in combination with direct osteolytic bone resorptive activity, caused a decrease in bone density^107^. In general, exposure to microgravity induces osteocyte apoptosis. When bones exposed to microgravity are examined, they are found to have more empty osteocyte lacunae than bones not exposed to microgravity. Furthermore, both the canilicular canals and the lacunae are larger, and the lacunae more irregular in shape in mice that undergo spaceflight compared to ground control animals, indicating that osteocytic osteolysis is occurring to a greater degree in the bone that undergoes unloading^90^. It also indicates that osteocyte morphology is varied during exposure to microgravity. The increase in empty lacunae, as well as change in size and lacunar shape, is attributed to an increase in osteocyte apoptosis and bone resorption^87,107^. This loss of osteocyte viability may dramatically alter bone and osteocyte mechanosensation during spaceflight. Additionally, osteocyte apoptosis, which precedes osteoclast recruitment during exposure to microgravity, results in upregulation of RANKL in neighboring osteocytes leading to large increases in osteoclast recruitment^108,109^. This results in greater bone resorption and decreased bone mass^108^. Changes in bone resulting from exposure to microgravity, such as osteocytic cell death and variations in lacunar shape and density, show similarities with known hallmarks of aged bone. Therefore, it is becoming more widely accepted that exposure to microgravity induces phenotypic changes in bone that resemble bone aging under standard gravitational conditions^87,110^.

HLS studies have corroborated in vitro observations that the genes encoding sclerostin and RANKL are upregulated when the bone is exposed to microgravity^93,111–113^. These HLS studies have also shown that increases in bone RANKL and decreases in expression of *Sost* are in part due to increased osteocyte apoptosis. For example, apoptotic osteocytes have been shown to release adenosine triphosphate (ATP) via pannexin1 channels, causing neighboring osteocytes to upregulate RANKL expression^114^. Similarly, a mouse model of osteoblast and osteocyte-specific Cx43 deficiency, with increased osteocyte apoptosis, is less responsive to the catabolic effects of simulated microgravity exposure^93,115^. Evidence suggests that bones from these mice have less sclerostin-mediated suppression of bone formation and less cortical osteoclast activity during unloading^93,115^. This suppression of normal osteocyte-driven bone resorption in osteocyte and osteoblast specific Cx43-deficient bone during prolonged unloading shows that Cx43 is a potential target to help prevent bone loss caused by exposure to microgravity.

## Osteoclasts

Osteoclasts, unlike osteocytes and osteoblasts are derived from HSCs^116^. Exposure to microgravity significantly modulates osteoclast number, viability, gene expression, and activity^2^. Under normal physiological conditions, osteoclasts resorb bone and suppress osteoblastogenesis through the secretion of specific factors. When exposed to unloading this effect is exacerbated, resulting in an increase in osteoclast activity and overall bone resorption while suppressing bone formation^9,60,116^. However, there are significant variations in osteoclast viability and function in response to microgravity, likely due to the different microgravity models and osteoclastic cell types used. Despite the significant bone loss observed in both in vitro and in vivo studies when osteoclasts are exposed to microgravity, a lack of understanding still exists regarding whether and how osteoclasts cell autonomously transduce changes in the mechanical environment and how this, in turn, regulates bone homeostasis.

## In vitro studies of the effects of microgravity on osteoclast cell physiology

Bone loss resulting from microgravity exposure is closely tied to variations in osteoclast activity. Sambandam et al. observed ~3000 differentially regulated genes in RAW 264.7 cells exposed to simulated microgravity using an RCCS^117^. There was significant upregulation of key genes encoding osteoclastogenic factors, specifically numerous matrix metalloproteinases and Cathepsin K (*Ctsk*)^117^. Along with the observed variation in gene expression, an increase in overall RNA concentration was also observed^118^. These observations suggest that not only does direct exposure to microgravity induce changes in osteoclast gene expression but microgravity also potentially increases osteoclast number and protein production. Upregulation of these key osteoclastogenic genes has also been linked to greater osteoclast differentiation of osteoclast progenitor cells when exposed to microgravity. For example, Tamma et al. seeded murine bone marrow mononuclear macrophages onto a bonelike biomaterial^118^. After 10 days of microgravity aboard the Foton-M3, increases in osteoclast size, osteoclast number, and a number of multinucleated cells were observed, indicating that exposure to microgravity induced greater osteoclastic differentiation. Additionally, increases in collagen telopeptides present in the media suggested an increase in osteoclast activity^118^. This microgravity-induced increased osteoclast activity was corroborated in, RAW264.7 cells^60^. For example, RAW264.7 cells exposed to microgravity produced a significant increase in resorption pit number and size^60^. Collectively, these data suggest that exposure to microgravity increases osteoclastogenesis, but the direct mechanism by which a lack of mechanical load causes these phenotypic changes is still relatively unknown.

Exposure to microgravity also sensitizes osteoclast precursors to soluble factors. Sexana et al. observed that brief 24-h exposure to microgravity sensitized osteoclast precursors to RANKL, which stimulates osteoclastogenesis^119^. Subsequent treatment with RANKL protein caused the formation of giant multinucleated osteoclasts and an increase in mRNA expression of genes encoding triiodothyronine receptor auxiliary protein (*Trap*) and *Ctsk*, indicating increased osteoclast maturation. Further RANKL-independent activation of the nuclear factor of activated T-cells, cytoplasmic 1 (NFATc1), extracellular signal-regulated kinase (ERK), and mitogen-activated protein kinase-p38 (p38MAPK) protein pathways was also observed. However, osteoclastogenesis was only enhanced with the addition of supplemental RANKL^119^. This suggests that despite the increased sensitivity to soluble factors, osteoclasts alone cannot activate mechanisms associated with increased osteoclastogenesis, as they do not secrete sufficient amounts of RANKL protein. Drastic changes in cytoskeletal structure and remodeling also occur in osteoclasts exposed to microgravity^60^. Though it is not fully understood how cytoskeletal changes mediate osteoclastogenesis, a correlative link between the two has emerged over the past 10 years.

### In vivo studies of the effects of microgravity on osteoclast cell physiology

Few studies have examined the effect exposure to microgravity has on osteoclastogenesis in vivo. The few studies that have corroborated the findings were found in vitro. Differential regulation of roughly 6000 genes in bone was observed in medaka fish aboard the ISS compared to ground control medaka fish^86^. Roughly 150 of those genes were differentially regulated between days 2 and 6 in fish aboard the ISS, indicating these genes are primarily involved in maintaining bone homeostasis in response to the mechanical loading environment^86^. In the same study, greater expression of tartrate-resistant acid phosphatase (TRAP+) cells in medaka fish exposed to microgravity suggests an increase in osteoclastogenesis as well, resulting in a significant decrease in mineral density of the pharyngeal bone and tooth region. In a different study, pre-osteoclastic cells isolated from C57/B6J female mouse femurs also showed an increase in differentiation potential after 15 days of spaceflight. This increase resulted in roughly 50% more multinucleated osteoclasts within the femur compared to femurs of ground control mice^51^. Osteoclastogenesis is in part regulated by RANKL, which is secreted by osteoblasts, which also secrete OPG, an inhibitor of RANKL. In a study by Lloyd et al., a single subcutaneous injection of OPG was given to C57BL/6J mice prior to 12 days of spaceflight^120^. This injection reduced bone loss from microgravity exposure to some degree. This was the result of a significant decrease in osteoclast number and a significant decrease in serum marker triiodothyronine receptor auxiliary protein-5 (TRAP5), a marker of osteoclastic activity^120^. However, the results of this study may not be definitive for multiple reasons. First, as noted by the authors, the mice used were not skeletally mature making it challenging to determine whether the effects observed were a result of microgravity or interference with the other cell populations responsible for growth, differentiation, and mineralization of the skeleton^120^. Second, the spaceflight was brief, and, as has been shown in other studies, osteoblastic and osteoclastic activity vary significantly within the first days of microgravity exposure^86,119^.

Numerous HLS studies have also shown a significant increase in osteoclast activity and number as well as a significant decrease in bone volume after 2–3 weeks of HLS^52,109,119,121^. The increased osteoclast activity results in an increased marrow cavity volume, decreased cortical thickness, increased trabecular porosity, and a weakening of bone mechanical properties^51,122^. This was further validated by Blaber et al., who reported female C57BL/6J mice flown aboard the STS-131 shuttle for 15 days had decreased bone volume fraction and diminished bone thickness^90^. Upon histological evaluation it was determined that a significant increase in TRAP+ osteoclast-covered bone surfaces was evident, suggesting that an increase in osteoclastic activity was, in part, responsible for the decrease in bone mass^90^.

## Skeletal Muscle Cells

Skeletal muscle contractions, unlike cardiac or smooth muscle contractions, are voluntary and act to generate the force on the skeleton for movement and postural support. At the cellular level, skeletal muscle is made up of mature muscle fibers, or myofibrils. Myofibrils are multinucleated myoblasts and can be some of the largest cells in the body, upwards of 100 microns in diameter and 30 cm in length in the human leg^123^. Each skeletal myofiber is made up of smaller units called myofibrils, which consist of actin (thin) and myosin (thick) filaments that overlap in a repeating structure^124^. These repeating structures called sarcomeres, comprised of actin and myosin, make up the main functional unit of skeletal muscle. The sarcomeres contract when Ca^2+^ is released from the sarcoplasmic reticulum, activated by nerve impulses, facilitating actin-myosin cross bridging and contraction^125^.

Individual skeletal muscle fibers can vary greatly between and within skeletal sites. Myofibers are typically classified based on the identification of myosin isoforms by histological staining for specific myosin ATPases, myosin heavy chain (MHC) constituents, and metabolic enzymes. Although not extensively reviewed here, there are over seven currently recognized myofiber types, ranging from the slowest contractile isoform (Type I ATPase, MHC I, oxidative) to the fastest contractile isoform (Type IIB ATPase, MHC IIx, oxidative)^126,127^. Skeletal muscles are made up of a hybrid of these individual muscle fibers but are usually classified based on their predominant fiber type. For example, the soleus is considered a slow-twitch oxidative skeletal muscle whereas the gastrocnemius is primarily fast-twitch and glycolytic. These histological and metabolic differences are important to note because they can lead to profound variations in the skeletal tissue and cellular response to disuse, which will be highlighted in subsequent sections.

### In vitro studies of the effect of microgravity on skeletal muscle cell physiology

In vitro studies have shed light on the molecular mechanisms in skeletal muscle cells regulated by direct exposure to microgravity. In vitro studies have the advantage of limiting the effects of systemic factors and allowing for examination of molecular mechanisms at the cellular level. Unfortunately, unlike in vivo studies, very few in vitro studies have been undertaken where isolated skeletal muscle cells are directly exposed to real microgravity conditions found during spaceflight. Several studies, although not recent, revealed severe and lasting phenotypic changes to skeletal muscle cells during short-term exposure to real microgravity (5–10 days). For example, primary rat L8 myoblasts, a nontumorigenic cell line, flown for 9 days aboard the STS-45 displayed an inability to fuse into myotubes, even upon returning to standard gravity (1 g)^128^. Another STS study, where avian skeletal muscle cells were seeded in tissue-engineered constructs, revealed a decrease in myotube cross-sectional area (−12%) and total protein synthesis (−75%) following 9 days of exposure to microgravity during spaceflight^12^. Interestingly these effects were augmented upon return to standard gravity after spaceflight, with concomitant increases in MHC and fibronectin protein. Finally, a more recent study by Uchida et al., showed that pre-differentiated L6 myoblasts cultured onboard the ISS for 10 days, had reduced myosin content (fast and slow), reduced myotube size, and increased oxidative stress compared to 1 g controls^129^. These important studies demonstrated that exposure to microgravity during spaceflight directly affects skeletal muscle cell differentiation and metabolism, suggesting skeletal muscle cells may be viable countermeasure targets^129^. However, the molecular mechanisms underlying these phenotypic changes remain poorly understood.

Ground-based analog studies examining skeletal muscle during simulated microgravity are more suited to study mechanisms of atrophy and have primarily used L6 rat wildtype^130^ or C2C12 dystrophic mouse^131^ myoblastic cell lines. Differentiation of these cells has been well characterized. For example, skeletal muscle cell differentiation, or myogenesis, from myoblasts requires the coordinated expression of four key muscle regulatory factors (MRFs). These include myogenic regulatory factor 5 (Myf5), myoblast determination protein 1 (MyoD), myogenin, and muscle regulatory factor 4 (Mrf4)^132–135^. For instance, Myf5 and MyoD are expressed in undifferentiated myoblasts and together are necessary for myoblast commitment^136,137^. Subsequently, myogenin and eventually MRF4 are highly expressed and aid in myoblast fusion and myotube formation^138^. One of the ways these MRFS are controlled is by exogenous signaling factors in the skeletal muscle microenvironment. For instance, exogenous IGF-1, which has been shown to cause skeletal muscle hypertrophy, upregulates myogenin via the PI3K/Akt signaling axis^139^. The P13K/AKT signaling axis has emerged as the predominant pathway controlling skeletal muscle metabolism. Reviewed extensively elsewhere^140^, in brief, the P13K/Akt pathway controls protein synthesis via mammalian target of rapamycin (mTOR) and glycogen synthase kinase 3β (GSK3β), and protein degradation via regulation of the transcription factors of the forkhead family of transcription factor (FOXO). Therefore, many recent ground-based in vitro studies have investigated the regulation of MRFs and P13K/Akt signaling components to understand how unloading directly affects skeletal muscle cell function.

Ground-based studies using a 3D clinostat, RPM, or RCCS have observed that exposure to simulated microgravity in C2C12 and L6 cells blocks myogenesis by decreasing MHC expression (fast and slow twitch), myotube thickness, and myotube fusion index^141–143^. These results suggest that simulated microgravity causes similar phenotypic changes in skeletal muscle as observed during spaceflight. Furthermore, these analog studies, due to the enhanced characterization available on earth, have suggested varying mechanisms for the decreased myogenesis. For example, Baek et al. observed that exposure to simulated microgravity decreases myogenesis by decreasing AKT phosphorylation and increasing phospholipase activity^141^. This resulted in the forkhead family of transcription factor O1 (FOXO1) stabilization and upregulation of genes associated with muscle degradation by apoptosis and autophagy^141^. This corroborates results by Harding et al. that showed exposure to simulated microgravity by RCCS increases expression of key skeletal muscle degradation pathways involving FOXO3 and muscle RING-finger protein-1 (MuRF1)^144^. Other results have demonstrated that simulated microgravity also impairs, via epigenetic changes, genes encoding downstream Akt targets, MyoD, and myogenin^129,145^. Overall, these results suggest that unloading directly affects the myogenic differentiation program by upregulating genes associated with degradation and downregulating genes associated with skeletal muscle differentiation and protein synthesis.

Another suggested mechanism by which exposure to microgravity may regulate skeletal muscle differentiation is through Ca^2+^ signaling. Studies have revealed that exposure to simulated microgravity via RPM decreases C2C12 myoblast [Ca^2+^]_i,_ but there are multiple proposed mechanisms by which this occurs. Damm et al. suggested that this was due to a decrease in levels of transient receptor potential cation channel subfamily C member 1 (TRPC1), a nonspecific plasma membrane cation channel^142^. The authors also showed that pharmacological inhibition of TRPC1 mimicked simulated microgravity effects by decreasing myoblast proliferation and terminal differentiation. Conversely, Calzia et al. showed that simulated microgravity by RPM induced increases in myoblast reactive oxygen species accumulation and this can be alleviated by intracellular Ca^2+^ release via pharmacological activation of Ryanodine receptors^143^. Ryanodine receptors play a vital role in skeletal muscle cell contraction by promoting sarcoplasmic reticulum Ca^2+^ release. Overall, these results suggest that dysfunctional calcium handling and influx may be a potential cause of decreased myogenesis and skeletal muscle metabolism resulting from exposure to simulated microgravity. Thus, promoting skeletal muscle calcium influx during microgravity exposure may be a key countermeasure for alleviating spaceflight-induced skeletal muscle loss. Next, we will review how microgravity has been shown to affect in vivo skeletal muscle from various preclinical and clinical studies.

### In vivo studies of the effect of microgravity on skeletal muscle

**Fig. 3:**
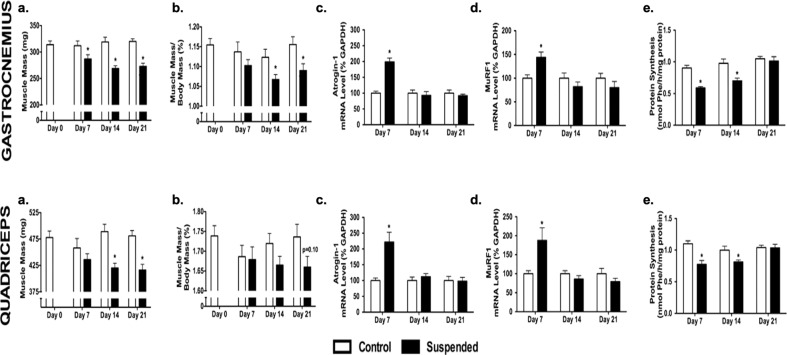
Skeletal muscle properties obtained from mice subjected to normal loading (Control) or mechanical unloading via hindlimb suspension (Suspended). **a** Total muscle mass, **b** muscle mass normalized to body mass, **c** mRNA levels of the gene encoding atrogin‐1, **d** mRNA levels of the gene encoding Murf1, and (**e**) protein synthesis rates were determined for both the gastrocnemius (top) and quadriceps (bottom). (*n* = 12–16/group).

Multiple animal species have been used to study how microgravity affects skeletal muscle development and function during spaceflight, though rodents have emerged as the predominate preclinical model. Exposure to spaceflight microgravity in skeletally mature mice and rats results in a rapid loss of soleus and gastrocnemius muscle mass within 2 weeks^146–150^. This is especially evident in oxidative slow-twitch muscles such as the soleus, compared to glycolytic fast-twitch muscles such as the extensor digitorium longus. Accompanying this decline in soleus muscle mass is a preferential loss of slow-twitch type I fibers and a gain in fast-twitch IIB fibers. Recent data have suggested that this is because fast-twitch muscles, unlike slow-twitch, upregulate protective signaling factors such as IGF-1, and interleukin 6 (IL-6), as well as stress-related genes encoding nuclear factor kappa-light-chain-enhancer of activated B cells (*NF-κB*) and heat shock protein family A member 1 A (*Hspa1a*)^146,147^. Furthermore, these same studies show degradation factors such as FOXO1 and Atrogin-1, part of the previously mentioned PI3K-kinase/AKT/mTOR pathway, are upregulated in skeletal muscles exposed to spaceflight microgravity. Exposure of skeletal muscle to unloading using ground-based analogs results in similar changes tissue-level changes seen during spaceflight. For example, skeletal muscle mass is dramatically decreased (15–20%) within the first weeks following HLS or neurectomy resulting from increased myostatin expression and decreased protein synthesis via the PI3K-kinase/AKT/mTOR pathway (Fig. 3)^52,151,152^. Surprisingly, unilateral casting or botox treatment during HLS can further exacerbate skeletal muscle mass and autophagy in the gastrocnemius but not the soleus^153,154^. Similar murine studies have also found evidence that skeletal muscle atrophy in these models is accompanied by an increase in key muscle E3 ubiquitin ligases, such as atrogin-1 and MuRF1, which promotes skeletal muscle degradation^155^. However, MuRF1 knockout mice have demonstrated resistance to skeletal muscle atrophy induced by denervation or HLS but not exposure to real microgravity^156–158^. This suggests that muscle atrophy resulting from simulated microgravity using ground-based analogs and skeletal muscle atrophy resulting from exposure to spaceflight may occur by divergent mechanisms that produce a similar tissue level phenotype.

In humans, exposure to microgravity during spaceflight causes rapid and preferential skeletal muscle volumetric loss and decreases in strength, predominately in the lower limb and trunk muscles. This is likely due to the role of these skeletal muscles in ambulation and postural support under standard gravity. Studies have shown rapid volumetric skeletal muscle loss, as revealed by magnetic resonance imaging (MRI), occurs in the quadriceps (−6%), gastrocnemius (−6%), and posterior back muscles (−10%) following 6-9 days of spaceflight^159–161^. Studies investigating the effects of short-term spaceflight on skeletal muscle using biopsies have demonstrated a preferential loss of fiber cross-sectional area depending on fiber type. For instance, preferential decreases in type II (fast-twitch) versus type I (slow-twitch) fiber cross-sectional area were observed in the soleus and vastus lateralis muscles between pre- and post-flight time points (5–10 days)^162,163^. Longer duration spaceflight missions abroad the ISS (~6 months) have resulted in greater skeletal muscle loss in the quadriceps (−12%), soleus-gastrocnemius (−13%), and posterior back skeletal muscles (−20%) resulting in a 25–30% loss of calf muscle twitch force^161,164,165^. This suggests a correlative effect between increased exposure to microgravity and skeletal muscle atrophy, regardless of muscle type. Overall, these data suggest that skeletal muscle mass rapidly decreases before becoming relatively stable after 3–4 months of microgravity exposure and are the result of decreased protein synthesis (~45% decrease) rather than degradation^161,166^.

## Integrative Physiology and Muscle-Bone Crosstalk

Microgravity and ground-based unloading models cause a host of systemic effects that can also directly affect the musculoskeletal system. These include immune cell dysfunction, cardiovascular deconditioning, neural/behavioral changes, lower limb hypoxia, and increases in endocrine factors such as glucocorticoid secretion^96,149,167–172^. Although beyond the scope of this review, it is important to note that these systemic changes also contribute to the negative health consequences of spaceflight and unloading on bone and skeletal muscle. Therefore, a more integrative physiological approach is necessary to combat this unique interplay between multiple organ systems. One way this is being realized is via the notion that direct biochemical cross-talk between bone and skeletal muscle is a major driver of these musculoskeletal changes, since these two distinct organs are in direct physical contact and share common developmental origins. This emerging concept has been referred to as muscle-bone crosstalk and support for this concept has been growing for the past decade. For example, factors produced by bone (osteokines) and regulated by unloading, such as Pge2 and RANKL, have anabolic and catabolic, respectively, effects on skeletal myogenesis, both in vivo and in vitro^84,173–175^. Conversely, many skeletal muscle factors (myokines) increased with unloading, such as IL-6 and myostatin, have detrimental effects on bone by supporting osteoclast formation and inhibiting osteoblast activity^176–178^. Furthermore, the myokine Irisin has been shown to alleviate bone and skeletal muscle loss resulting from HLS when given exogenously^179,180^. These results suggest that targeting shared biochemical pathways between skeletal muscle and bone may be efficacious in ameliorating musculoskeletal defects associated with unloading and spaceflight. However, these biochemical crosstalk mechanisms are complex, and progress has been hindered by the fact most researchers, until recently have not examined the response of bone and muscle as a unit to unloading. Potential therapies as well as their efficacies for augmenting skeletal muscle and bone loss are presented next.

## Microgravity Countermeasure Development

Correcting the homeostatic imbalances resulting from exposure to microgravity in both bone and skeletal muscle remains an important area of research. Most pharmacological approaches target pathways specific to either bone or muscle tissue, while exercise and nutritional treatments target both tissues, as well as other distant organ systems.

Pharmacological treatments for bone loss resulting from exposure to microgravity are becoming more prevalent as clinical treatments for osteoporosis expand and our understanding of how bone maintains homeostasis improves^181^. Many of the countermeasure targets to augment bone loss due to osteoporosis and spaceflight have initially focused on limiting resorption. Bisphosphonates and anti-RANKL therapies (Denosaumab and OPG-Fc) work directly on bone by limiting the formation and action of bone-resorbing osteoclasts. These therapies currently remain a cost-effective and potent treatment for osteoporosis and have shown efficacy in studies of hindlimb unloading and spaceflight^182^. For example, treatment of mice with bisphosphonates during HLS preserved bone mass by limiting bone resorption and by transiently stimulating bone modeling^183,184^. In addition, growing female mice given a single treatment of OPG-Fc antibody (20 mg/kg), a RANKL inhibitor, displayed attenuated bone loss during 12 days of spaceflight aboard STS-108.^120^ Importantly, the use of similar antiresorptive agents in astronauts have yielded promising results when used during spaceflight. For instance, seven astronauts receiving weekly oral bisphosphonates (alendronate) and combined exercise treatments (treadmill, cycle ergometer, and a resistance exercise device (aRED)) displayed no bone loss, as assessed by bone densitometry (DXA) or quantitative computed tomography (QCT), whereas those who only exercised displayed bone loss.^185^. This suggests that short-term bisphosphonate treatment, at least with exercise, can protect astronauts from bone loss in space. Denosumab, the human equivalent to OPG-Fc and used to treat osteoporosis, has yet to be tested in astronauts^186^. More long-term studies (>6 months) with the use of these antiresorptive agents alone during spaceflight are needed to directly determine their long-term safety and efficacy for reducing bone fracture risk.

Anabolic agents, which primarily work to promote bone mass by stimulating osteoblast formation and activity, are also potential countermeasures for bone loss resulting from unloading and spaceflight. One of the primary pathways targeted by these therapies is the WNT/ß-catenin pathway. As reviewed extensively elsewhere^187,188^, substantial evidence suggests that WNT/ß-catenin pathway activation promotes osteoblast cell lineage differentiation and survival while indirectly inhibiting osteoclast activity. Thus, targeting WNT/ß-catenin could potentially mitigate bone loss during unloading and spaceflight. As mentioned earlier in this review, sclerostin, an inhibitor of WNT/ß-catenin signaling, is upregulated in bone cells after exposure to microgravity and elevated in the serum of individuals on extended bedrest^2,96,103,189^. Recently, a human sclerostin neutralizing antibody, Romosozumab, was approved for osteoporosis treatment^190^. Furthermore, this same biologic attenuated mouse bone loss resulting from HLS or spaceflight (STS-135)^191,192^. Interestingly, Spatz et al. showed that in mice undergoing HLS, neutralizing sclerostin antibody preserved hindlimb bone mass proportional to the amount of unloading in their partial weight-bearing model^191^. Furthermore, they showed that anti-sclerostin antibodies had no effects on preserving skeletal muscle mass. These results suggest that sclerostin antibody treatment should be combined with exercise in NASA astronauts for maximal effect. Although promising, Romosozumab has resulted in side effects, such as an increased risk of cardiovascular disease^10^, and therefore more long-term studies on its use on Earth and during spaceflight are needed.

Another promotor of WNT/ß-catenin that has shown promise in mitigating bone loss with unloading is neural EGF-Like Protein 1 (NELL-1). James et al. showed that recombinant NELL-1 (rhNELL-1) induces WNT/ß-catenin activation via the ß1 integrin in osteoblast precursors and osteoclasts in vitro and can prevent ovariectomy-induced bone loss in mice^193^. Follow-up work by this group showed that increasing the half-life and bone retention of rhNELL-1 by PEGylation and inactive bisphosphonate-linkage can improve bone fracture healing in ground control mice and mitigate bone loss in mice undergoing spaceflight^194,195^. Clinical evaluation, including long-term follow-up, of rhNELL-1 in osteoporotic patients on Earth, is needed before this treatment can be examined in astronauts.

Other bone loss therapies currently investigated during unloading and spaceflight include bone morphogenic proteins (BMP’s), diet, and exercise regimens. The most commonly used BMP, BMP-2, works by binding multiple transcription factors that upregulate genes such as *Runx2* and the gene encoding cyclooxygenase-2 (*Cox2*)^196,197^. Additionally, BMPs activate the MAPK pathway and its downstream effectors ERK1/2 and p38^198,199^. Supplementation with BMPs in mouse models has also been shown to partially alleviate bone loss after exposure to microgravity, but their efficacy in humans is complicated by reports of ectopic bone formation and increased cancer risk^200–203^.

Exercise and nutritional therapies have shown only moderate protection from bone loss related to microgravity exposure, with improvements in training regimens and the equipment available to subjects playing a large role in the success of the therapy^10,204,205^. Astronauts aboard the ISS used the interim Resistive Exercise Device (iRED) until 2004, and now its newer higher loading counterpart (up to 600 pounds), the advanced Resistive Exercise Device (aRED) to perform resistive strength exercises^206,207^. Although the use of the aRED and adequate nutrition (Vitamin D) can boost bone formation indices to partially offset the enhanced resorption in space^3^, as mentioned previously, only use of an antiresorptive with exercise can effectively limit bone loss following 4–6 month ISS stays^185^. Limiting bone loss early and to a high degree is paramount since follow-up DXA and QCT show differential recovery of bone mass up to 1–2 years post-flight^208,209^. Importantly, long-term changes such as permanent loss of femoral trabecular bone volume fraction and thinner cortices cause irreversible changes to the bone that may affect long-term fracture risk upon return to Earth or during future spaceflight.

Skeletal muscle in astronauts undergoing spaceflight shows significant atrophy and strength deficits in just a few weeks. As mentioned earlier, this has been primarily due to unloading-induced decreases in protein synthesis. Protein synthesis and metabolism are positively controlled via the PI3K-Akt-mTOR pathway^140,210,211^. In this pathway, IGF-1 activates AKT phosphorylation and mTOR activation, thereby inducing skeletal muscle hypertrophy. In contrast, the soluble inhibitor myostatin, binds to activin receptor type 2, blocking mTOR activity thereby promoting skeletal muscle atrophy. Furthermore, the aforementioned work in this review has shown that real and simulated microgravity upregulate myostatin expression but decreases IGF-1 expression in skeletal muscle^141,142,146,212^. Therefore, exogenous IGF-1 and neutralizing antibodies targeting myostatin have emerged as viable countermeasure targets for mitigating skeletal muscle loss due to spaceflight.

Initially, IGF-1 overexpression in skeletal muscle by gene transfer or in transgenic mice showed promise in mitigating skeletal muscle degeneration and atrophy due to glucocorticoid use and injury in mice and rat models^213,214^. However, more recent results using similar approaches during immobilization by casting or HLS have yielded fewer promising results. For example, although overexpression of IGF-1 during murine development increased skeletal muscle size and contractile force at baseline, it failed to prevent significant atrophy with disuse^213,215^. The use of exogenous IGF-1 was also tested during 10 days of spaceflight in mice flown on STS-77^216^. Although IGF-1’s influence on spaceflight-induced skeletal muscle atrophy was not reported, it did increase femoral bone mass and strength by increasing periosteal bone apposition.

In contrast to IGF-1, recent results using myostatin inhibitors have yielded more consistent outcomes regarding skeletal muscle atrophy resulting from unloading and spaceflight. For example, weekly treatment of young mice with REGN1033 (a monoclonal antibody against myostatin) eliminated tibialis anterior and gastrocnemius muscle mass changes during casting and attenuated losses with hindlimb unloading^217^. Similar effects have been reported for skeletal muscle loss in mice and rats exposed to spaceflight. For instance, Smith et al. demonstrated that the use of myostatin inhibitor antibody (YN41), as part of the rodent research-3 mission, alleviated spaceflight-induced hindlimb skeletal muscle atrophy and blocked decreases in grip strength following 6 weeks aboard the ISS^218^. However, myostatin inhibition had no effect on decreases in trabecular BMD. On the other hand, a recent study has shown that using a dual systemic inhibitor of myostatin and activin A ligand signaling can rescue bone and skeletal muscle loss in wild-type mice following 33 days aboard the ISS.^219^. Lee et al. showed that using this broader ligand targeting approach, lean mass, hindlimb skeletal muscle mass, and femoral BMD were greater in wild-type mice undergoing 33 days of spaceflight compared to age-matched ground control animals. Importantly, age-matched myostatin knockout mice undergoing the same spaceflight regimen, while displaying skeletal muscle protection against spaceflight-induced atrophy, did not show protection against spaceflight-induced decreases in BMD. This suggests that the targeting of activin A and myostatin collectively is a more promising approach to protect against bone and muscle loss in response to spaceflight-induced microgravity.

Finally, increasing effort has been placed on examining the role of nutrient supplementation and resistive exercise in order to maintain skeletal muscle mass during exposure to microgravity. Unfortunately, the use of the iRED and aRED devices has not been able to ameliorate skeletal muscle mass and strength deficits during shorter and longer duration spaceflight^164,181,220^. In contrast, bedrest analog studies using amino acid supplementation and/or rehabilitative aerobic and resistive exercises similar to that used on the ISS, have shown the ability to prevent skeletal muscle loss due to lack of mechanical loading^221–224^. Skeletal muscle atrophy and functional impairments are more of a concern for ensuring that NASA crewmembers have optimal dexterity, metabolism, and strength capacity for performing in-flight or EVA-based tasks. This is because, upon return to earth, most skeletal muscles, except for some paraspinal muscles, return to their normal composition and size^225,226^.

It is likely that treatment with both pharmacological agents combined with exercise and nutritional therapy will best treat the catabolic and anabolic changes observed in bone and muscle tissue during exposure to microgravity. In addition, the synergy between the two tissue systems should not be ignored.

## Conclusion

In the near future, NASA hopes to enable the return of humans to the moon and prepare for manned missions to Mars. For these missions to be successful, humans will need to be prepared for long-term space exploration and be equipped to handle deep space stressors, such as microgravity. As discussed in this review, exposure to microgravity causes severe negative health effects on the musculoskeletal system. Musculoskeletal tissue is heavily dependent and adaptive to its mechanical loading environment. The lack of mechanical loading of the musculoskeletal system that occurs during exposure to microgravity results in skeletal muscle atrophy and loss of bone mass, putting astronauts at risk for injury during spaceflight and upon normal gravitational reloading.

This review has detailed the current understanding of how exposure to microgravity effects the musculoskeletal system, but it has also revealed gaps in our current understanding that still need to be addressed. With recent evidence revealing the importance of bone and muscle crosstalk, future studies will need to focus on how exposure to microgravity affects the interaction of these tissue systems. Additionally, further interactions between the musculoskeletal system and the endocrine and nervous system need to be evaluated but the implications of microgravity exposure on these systems was not within the scope of this review. Moreover, how the musculoskeletal system responds to reloading after extended periods of exposure to microgravity is also of significant interest. There are still questions as to how much force the musculoskeletal system can generate after exposure to microgravity before sustaining injury. This information will also allow for more effective countermeasures to be developed, mitigating the catabolic effects microgravity exposure has on the musculoskeletal system. These avenues will provide greater insight into how exposure to microgravity affects the musculoskeletal system, its effects on musculoskeletal ability, and how we can counteract the negative effects.

## Data Availability

No original data was created for this manuscript. For data, please contact the authors of such data.
